# The challenge of concomitant infections in the coronavirus disease 2019 pandemic era: Severe acute respiratory syndrome coronavirus 2 infection in a patient with chronic Chagas disease and dimorphic leprosy

**DOI:** 10.1590/0037-8682-0504-2020

**Published:** 2020-11-06

**Authors:** Patricia Shu Kurizky, Selma Regina Penha Silva Cerqueira, Débora Vilela Cunha, Cleandro Pires de Albuquerque, Rodrigo Barbosa Aires, Licia Maria Henrique da Mota, Ciro Martins Gomes

**Affiliations:** 1 Hospital Universitário de Brasília, Serviço de Dermatologia, Brasília, DF, Brasil.; 2 Universidade de Brasília, Faculdade de Medicina, Pós-graduação em Ciências Médicas, Brasília, DF, Brasil.; 3 Universidade de Brasília, Faculdade de Medicina, Laboratório de Dermatomicologia, Brasília, DF, Brasil.; 4 Hospital Universitário de Brasília (HUB), Serviço de Reumatologia, Brasília, DF, Brasil.; 5 Universidade de Brasília, Núcleo de Medicina Tropical, Brasília, DF, Brasil.

**Keywords:** Coinfections, COVID-19, Leprosy, Chagas disease

## Abstract

Coronavirus disease 2019 (COVID-19) was first officially described in Brazil on February 26^th^, 2020. The accumulation of reports of concomitant infections with severe acute respiratory syndrome coronavirus 2 (SARS-CoV-2) and pathogens that cause diseases endemic to tropical countries, such as dengue and chikungunya fever, has started to draw attention. Chagas disease and leprosy remain public health problems in many developing countries, such as Brazil. In this manuscript, we describe a case of concomitant leprosy, Chagas disease, and COVID-19, highlighting the cutaneous manifestations of SARS-CoV-2 infection and the clinical behavior of household contacts who previously received prophylactic Bacillus Calmette-Guérin vaccines.

## INTRODUCTION

Coronavirus disease 2019 (COVID-19) was first officially described in São Paulo, Brazil on February 26^th^, 2020, and since then, the country has experienced a concerning increase in the incidence of COVID-19. As of June 10^th^, 2020, 772,416 positive cases had been identified, with 39,680 deaths^1^. The growing number of reports of concomitant infections with SARS-CoV-2 and pathogens that cause diseases endemic to tropical countries, such as dengue and chikungunya fever, has drawn attention^2^. Chagas disease and leprosy continue to remain public health problems in many developing countries, including Brazil^3^. In this manuscript, we describe a case of concomitant leprosy, chronic Chagas disease, and COVID-19, highlighting the cutaneous manifestations of SARS-CoV-2 infection and the clinical behavior of household contacts who had previously received prophylactic Bacillus Calmette-Guérin (BCG) vaccines. Patient inclusion was approved by the Ethics Committee of the Faculty of Medicine of Universidade de Brasília after informed consent was obtained (95411718.2.0000.0030).

## CASE REPORT

**FIGURE 1:**
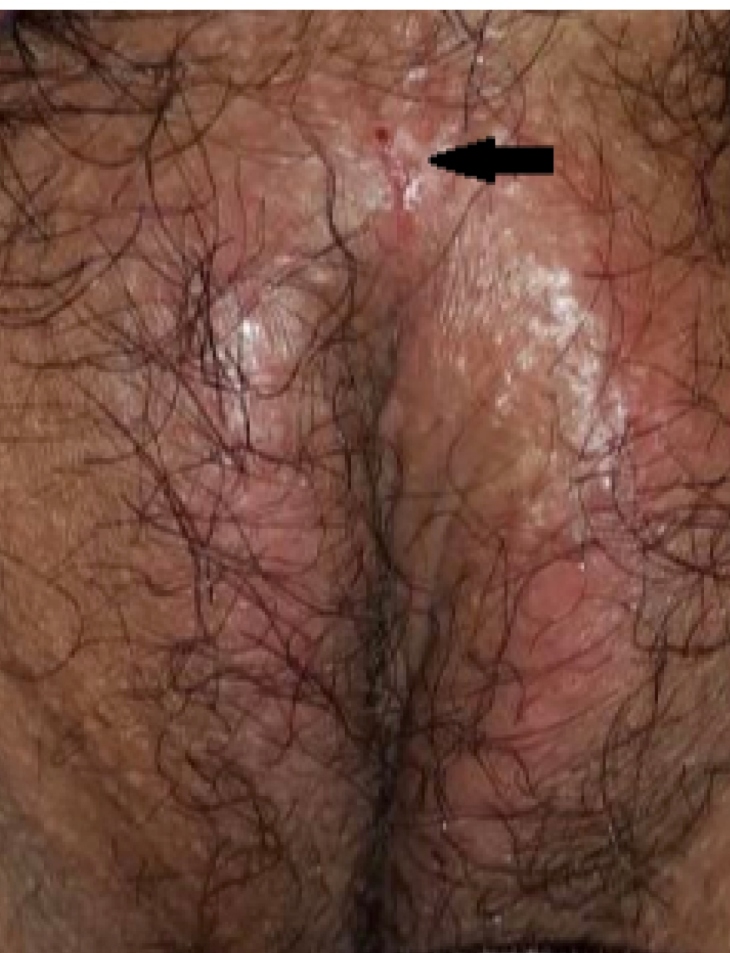
Ulcerated vulvar lesion.

The patient, a 43-year-old female resident of Paranoá, in the Federal District of midwestern Brazil, was diagnosed with dimorphic leprosy in 2010 via positive smear microscopy and the presence of asymmetric multiple mononeuritis. She started treatment with multibacillary multidrug therapy (MDT-MB) in August 2010. However, she experienced multiple interruptions in treatment due to adverse reactions, until the therapy was finally terminated in April 2020 after the administration of dapsone caused hemolytic anemia. She was also diagnosed with Chagas disease in 2013 via serology (chemiluminescence), and because she had cardiac involvement and arrhythmia, she required a pacemaker as well as treatment with propranolol (80 mg/day) and Marevan (5 mg/day). The patient was scheduled to start a monthly treatment regimen with rifampicin, ofloxacin, and minocycline. However, she returned in May 2020 with complaints of fatigue, odynophagia, anosmia, ageusia, and headache for the prior 3 days. Reverse transcriptase polymerase chain reaction (RT-PCR) for SARS-CoV-2 was performed from a nasopharyngeal swab sample, and the result was positive. Her condition progressed, and she developed diarrhea and rectal bleeding in addition to cheilitis and painful, ulcerated lesions on her genital (Figure 1) and oral mucosae; however, the patient did not experience worsening of her leprosy symptoms, nor did she develop reactional states. No fever or respiratory symptoms were observed. The patient lived with her husband, who was also using MDT-MB treatment for leprosy, and her daughter, who had received BCG vaccine chemoprophylaxis in January 2020. Although her husband and daughter were classified as close household contacts, neither presented with symptoms, nor did they test positive for SARS-CoV-2 using RT-PCR or anti-SARS-CoV-2 antibodies using serology.

## DISCUSSION

Leprosy is a chronic, infectious, granulomatous disease that affects the skin and peripheral nerves and is caused by *Mycobacterium leprae* and *Mycobacterium lepromatosis*. The majority (90%) of current leprosy cases are found in Brazil, India, Nepal, Myanmar, Madagascar, and Mozambique, and 80% of the cases that occur in the Americas are concentrated in Brazil^4^. There are no current data regarding cases of concomitant COVID-19 and leprosy. However, information about the possible effects of these concomitant diseases and the effects of the drugs used for the treatment of either COVID-19 or leprosy may be important. Another important focus is the possible increase of the frequency and intensity of the reactional state, as would be expected in cases in which leprosy patients contract other viral infections^5^. Leprosy reactions are characterized by malaise and the exacerbation of preexisting lesions, with subsequent erythema and pain that are usually accompanied by neuritis and severe edema of the extremities, occurring in 40% of borderline lepromatous patients^5^.

In the present case, the patient was in the chronic phase of Chagas disease, characterized by congestive cardiac failure, arrhythmia (not controlled by drugs), and right bundle branch block, which were treated with a pacemaker, propranolol, and an anticoagulant^6^.

Recent data from the COVID-19 pandemic have shown that the virus can affect the cardiovascular system. The damage due to COVID-19 is probably multifactorial, and can result from an imbalance between high metabolic demand and low cardiac reserve, systemic inflammation, and/or thrombogenesis. Direct cardiac damage from the virus also occurs, primarily in patients with cardiovascular risk factors or preexisting cardiovascular diseases^7^.

Although the patient was classified as having borderline leprosy and Chagas cardiomyopathy, she did not experience a recrudescence of leprosy symptoms, worsening of previous cardiovascular manifestations, or a severe case of COVID-19. Although the vast majority of patients with COVID-19 have a good clinical outcome, we hypothesize that leprosy treatment, due to its immunomodulatory properties, could also have protected this patient. Moreover, exposure to *M. leprae* itself, due to its immunogenic similarities to *M. bovis*, could conceivably confer protection against the severe manifestations of COVID-19, given that the BCG vaccine has already been postulated to possess such properties^8^. These hypotheses are worth testing directly in future studies.

The present patient also developed mucosal manifestations, likely related to SARS-CoV-2 infection, as reported elsewhere. Initial studies from China reported low frequencies of cutaneous manifestations in COVID-19 patients (0.2%), but reports are increasing, including acro-ischemia, chilblain-like eruptions, petechiae or purpuric rash, chickenpox-like rash, urticaria, erythema multiforme, maculopapular rash, mottling, and pityriasis rosea-like rash^8^. Ulcerative mucosal lesions, similar to those seen in our patient, were also described by Carreras-Presas et al^9^. These mucosal ulcers are common primary lesions observed in other viral infections, such as herpes and hand, foot, and mouth disease. It is worth noting that our patient had no fever or cough but initially presented with mucosal manifestations.

It is important to highlight the hypothesis raised by the scientific community that the BCG vaccine may mitigate the effects of COVID-19, as it could explain why the patient’s daughter did not contract COVID-19 despite being in close contact with her. Recent studies have demonstrated an inversely proportional relationship between the number of COVID-19 cases in countries with widespread BCG vaccine use and the relatively high numbers of COVID-19 cases in countries that have suspended the universal administration of the BCG vaccine, such as Italy, Spain, and the United States^10^. Interestingly, in Brazil, the BCG vaccine, in addition to being standard for newborns, is also used as prophylaxis for leprosy in household contacts. It is possible that a specific sub-analysis of this particular coinfection could add further information about the natural history of COVID-19 and leprosy.
